# Multiparameter Evaluation of the Heterogeneity of Circulating Tumor Cells Using Integrated RNA *In Situ* Hybridization and Immunocytochemical Analysis

**DOI:** 10.3389/fonc.2016.00234

**Published:** 2016-11-08

**Authors:** Yongqi Wu, Kyoung-Joo Jenny Park, Clayton Deighan, Peter Amaya, Brandon Miller, Quintin Pan, Maciej Zborowski, Maryam Lustberg, Jeffery Chalmers

**Affiliations:** ^1^William G. Lowrie Department of Chemical and Biomolecular Engineering, The Ohio State University, Columbus, OH, USA; ^2^Department of Biomedical Engineering, The Ohio State University, Columbus, OH, USA; ^3^Department of Otolaryngology-Head and Neck Surgery, The James Cancer Hospital and Solove Research Institute, Wexner Medical Center at The Ohio State University, Columbus, OH, USA; ^4^Department of Biomedical Engineering, Cleveland Clinic, Cleveland, OH, USA; ^5^Breast Medical Oncology, Department of Internal Medicine, James Cancer Hospital, Ohio State University Comprehensive Cancer Center, Columbus, OH, USA; ^6^Analytical Cytometry Shared Resource, Ohio State University Comprehensive Cancer Center, Columbus, OH, USA

**Keywords:** circulating tumor cells, negative magnetic enrichment, multiparameter analysis, integrated RNA ISH and ICC staining, fluorescent labeling

## Abstract

Circulating tumor cells (CTCs) are routinely identified as cytokeratin (CK)-positive, epithelial cell adhesion molecule (EpCAM)-positive, and CD45-negative and are enriched based on EpCAM. However, there are a number of methodological challenges regarding both isolation and characterization of these rare CTCs including downregulation or absence of EpCAM in a variety of solid tumors leading to the omission of subpopulations of CTCs, difficulties in analyzing RNA and protein targets in CTCs due to the rarity of these cells, and low levels of targets and technological limitations of visualizing the targets of interest on each individual cell. Building on our previous CTC research on CD45-based negative magnetic separation and four-color fluorescent immunocytochemical (ICC) staining, RNA *in situ* hybridization (ISH) was applied to fluorescently target mRNA sequences corresponding to tumor-related genes at the single CTC level. Multiple categories of markers are targeted including CK, human epidermal growth factor receptor family markers, Hedgehog pathway markers, human papillomavirus markers, and protein arginine methyltransferase 5. In addition, an integrated method of RNA ISH and fluorescent ICC staining was developed to visualize CTCs on both mRNA and protein levels. The robustness of the integrated co-ICC and RNA ISH staining was demonstrated by a series of tests on representative tumor markers of different categories. The integrated staining can incorporate the advantages of both RNA ISH and fluorescent ICC staining and provide more intense signals and more specific bindings. With this integrated staining methodology, distinct staining patterns were applied in this report to facilitate the searching and characterization of rare subgroups of CTCs. These results support the existence of diverse groups of CTCs at both protein and mRNA transcript levels and provide an analytical tool for the research on CTCs of rare subgroups.

## Introduction

Circulating tumor cells (CTCs) are tumor cells or tumor-related cells that are shed into human circulatory systems and areas associated with cancer metastasis. They have the potential to serve as liquid biopsy material that can provide a wealth of real-time information on patient disease state, treatment efficacy, prognosis, and potential drug targets. This is particularly helpful due to the challenges in obtaining serial metastasis biopsies and to distinguish the differences between metastasis and primary tumor biology. Although this idea is promising, the field has faced multiple challenges in the isolation and characterization of CTCs that have impacted the broad acceptance of the utility of these circulating cells (1, 2).

The most common method to identify and enumerate CTCs is through a positive selection by targeting the epithelial cell adhesion molecule (EpCAM). However, the EpCAM-based positive selection brings significant challenges in using CTCs as diagnostic and prognostic tumor markers, or as predictive markers for developing therapeutic drug targets, since more than one-third of patients with metastatic diseases do not have detectable CTCs by EpCAM-based technologies (3). In fact, a number of studies have shown that EpCAM-negative cells, presumably CTCs, are present in cancer patients (4, 5). In addition, Zhang et al. (6) showed that the cells of EpCAM-negative phenotype can establish tumors in brain and metastasize to lung in nude mice, clearly demonstrating the tumorigenicity of this phenotype. Furthermore, Lustberg et al. (7) demonstrated that the elevated level of a CD45−, EpCAM−, CK+ phenotype is positively correlated with a decreased overall survival. One hypothesis is that part of CTCs from this subset of the patients have undergone epithelial-to-mesenchymal transition (EMT), resulting in downregulation of EpCAM and other epithelial-associated markers. To avoid the potential bias associated with positive selection methods, we have developed our negative magnetic enrichment (NME), in which we enriched CTCs by removing red blood cells (RBCs) through lysis and by removing CD45-positive cells through magnetic labeling and separation (8–10).

Fluorescent immunocytochemical (ICC) staining is typically used to analyze CTCs with multiple tumor-related markers, including cytokeratin (CK), human epidermal growth factor receptor (HER) family markers, stem cell markers, etc. (11, 12). Admittedly, as ICC staining targets functional proteins, it can provide the most direct information with respect to the cells. However, ICC staining is not always definitive with respect to protein expression levels. For some common tumor markers, it is not easy to set the proper “cut-off” to determine positive signals from negative ones in a CTC using ICC staining. Furthermore, non-specific binding, which is reported to be primarily caused by the binding of primary and secondary antibodies to endogenous Fc receptors, raises the potential of false positive ICC signals (13). As an alternative to ICC staining, RNA analysis is a common approach to provide complementary information about various tumor-related markers.

The prevailing way to perform mRNA-level analysis on CTCs is to use RT-PCR or microarray analysis on the extracted mRNA from the CTCs that are isolated by oncomarker-based positive selection (14–16). Most mRNA analysis with RT-PCR and microarrays, nevertheless, can only analyze the mRNA isolated from CTCs in an aggregated (averaged) form without giving specific information about the presence of mRNA signals at a single-cell level. To analyze the heterogeneity of CTCs, some microchip-based single CTC capturing devices have made it possible to separate out individual CTCs in fluid drops by targeting CTC surface markers (17, 18). Meanwhile, with the capability of capturing individual CTCs, recent progress in single-cell mRNA and transcriptome profiling provides the transcription information of certain oncogenes and transcriptome library for single CTCs from some cancer types (19). However, the throughput of these cutting-edge single-cell assays is relatively low; furthermore, these assays do not provide information regarding the morphology of the CTCs and the locations of certain proteins and mRNAs within the CTCs.

To facilitate RNA analysis of CTCs at single-cell resolution with direct visualization of targets, fluorescent *in situ* hybridization (ISH) on mRNA is a viable alternative to RT-PCR or microarray analysis. RNA ISH is a visualized technique to fluorescently label a specific nucleic acid sequence on mRNA with short anti-sense nucleic acid sequences. Dynamic mRNA transcription and movement research shows that the quantification and timeliness of RNA ISH are adequate to track the appearance and the location of single mRNA molecules (20). Furthermore, in contrast to the use of antibodies in which the only information known is the name of the specific clone (not the actual protein sequence targeted, binding pattern, etc.), the probe sequence and target sequence of RNA ISH are precisely known and have only 20 bp oligonucleotides in the most recently reported versions (21, 22).

In this study, we captured CTCs by our NME methodology, in which the only assumption is that CTCs do not have high expression of CD45. The isolation procedure with our NME can retain most heterogeneous CTCs, including the CTCs that are negative in epithelial markers. Meanwhile, we established a method to perform integrated RNA ISH and ICC staining on the rare cells isolated from the blood samples of the patients with advanced malignancies. We tested the robustness of the method in various categories of tumor markers, including HER family markers, epithelial markers, Hedgehog pathway markers, and enzymatic markers, on cell lines and isolated CTCs from different tumor origins. We further incorporated RNA ISH staining of human papillomavirus (HPV) viral mRNA with the integrated RNA ISH and ICC staining of tumor markers in CTCs. In this study, a comprehensive analytical tool for heterogeneous rare CTCs was established to enrich CTCs and visualize them on both mRNA and protein levels with a variety of tested markers.

## Materials and Methods

### Cell Culture

Breast cancer cell lines, MCF-7 (HTB-22) and BT474 (HTB-20), and tongue cancer cell line, SCC-4 (CRL-1624), were procured from ATCC. HPV-positive cell lines, SCC-47 (UM-SCC-47) and SCC-90 (SCC-090), were provided by Dr. Quintin Pan’s lab, Department of Otolaryngology, The Ohio State University. SCC-47 was originally acquired from EMD Millipore, whereas SCC-90 cell line was originally created by Dr. Gollin’s lab with previously reported source and protocol (23). Hedgehog pathway active cell line, T47D (HTB-133), was provided by Dr. Majumder’s lab, College of Medicine, The Ohio State University, and was originally acquired from ATCC. These cells were grown to mid-log phase in Dulbecco’s modified Eagle medium (DMEM) (Cellgro) with 10% fetal bovine serum (FBS) (Invitrogen) and 1% non-essential amino acid (Cellgro) at 37°C in 5% CO_2_ atmosphere. Cell lines were harvested by washing the adherent cells with phosphate-buffered saline (PBS) and subsequently incubating with Accutase (Innovative Cell Technologies, Inc.) for 10 min at 37°C to detach cells from the culture flasks. Accutase was then neutralized with the culture medium before pelleting the cells by centrifuging at 350 × *g* for 5 min. Cells were finally resuspended in culture medium for downstream experiments.

### Patient Samples and Blood Collection

Patients with various metastatic solid tumor malignancies, older than 18 years of age, were enrolled in Institutional Review Board (IRB)-approved protocols that permit CTC characterization of blood specimens. All patients gave their informed consent to participate in the study. After several standard blood tubes were drawn for routine chemotherapy labs, either prior to initiation of a new line of systemic therapy or at progression, peripheral blood (7.8–17.7 mL) was collected in BD Vacutainer Heparin tubes (BD Biosciences) for CTC enumeration and processed within 4 h of blood collection. The clinical characteristics of patients are summarized in Table S1 in Supplementary Material.

### Normal Control Blood Collection

Healthy donor blood was collected from volunteer donors after obtaining informed consent using an IRB-approved protocol at The Ohio State University Medical Center and was processed in the same manner as tumor patient blood samples. The protocol was approved by the Cancer Institutional Review. Source leukocytes (buffy coat), also as a control, were purchased from American Red Cross, Central-Southeast Ohio region.

### Sample Processing for Negative Depletion Enrichment

**Figure 1:**
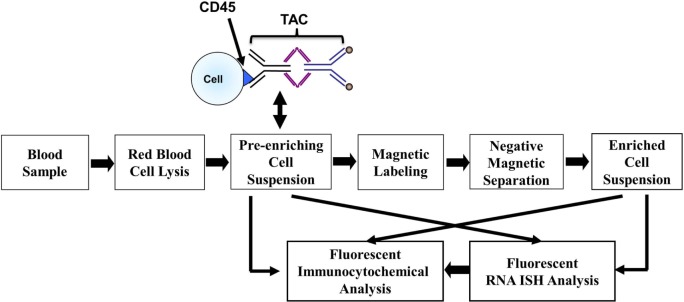
**Workflow chart of CTC magnetic enrichment, RNA ISH, and fluorescent immunocytochemical staining**.

Blood samples were kept at ambient temperature and subjected to a CTC enrichment procedure within 4 h after blood collection using our NME approach, as described previously (24, 25). Briefly, blood samples were subjected to an optimized RBC lysis step and were labeled with anti-CD45 tetrameric antibody complex (TAC; Stem Cell Technologies). The cells with CD45 TAC were then incubated with dextran-coated magnetic nanoparticles and subsequently ran through the quadrupole magnetic sorter (QMS) system. The CD45 negative or low positive cells from the blood of patients or healthy donors enriched in the NME, as described above, is referred as NME cells in the following parts. The effectiveness of NME on patient blood samples was evaluated by the log_10_ of the ratio between the total nucleated cell number before NME and that after NME. Figure 1 shows the overall separation and analysis flow chart.

Nucleated cell counts were determined prior to RBC lysis, after RBC lysis, and after NME. Lysis efficiency, nucleated cell log depletion, and total cell log depletion were used to evaluate the performance of the NME process. The calculation for these three parameters was introduced in our earlier publication (10). As previously reported, over 120 blood specimens from breast cancer patient have been processed with this methodology. The average nucleated cell log depletion, average total cell log depletion, and average lysis efficiency were 2.56, 5.63, and 77.0%, respectively.

### RNA *In Situ* Hybridization Analysis

Approximately 2 × 10^4^ NME cells from the blood of a patient or healthy donor were used for each RNA ISH slide. Approximately 2 × 10^4^ source leukocytes spiked with 5 × 10^3^ cells from a cancer cell line were applied on each control slide. Cell suspensions were spun onto superfrost plus slides (Fisher Scientific) and fixed by 4% paraformaldehyde for 10 min. Subsequently, the cells were treated with QuantiGene ViewRNA ISH Cell Assay Kit (Affymetrix), following the manufacturer’s protocol. Different nucleic acid probes with type 1, type 4, and type 6 fluorescent labeling conjugations were used to detect the corresponding mRNA sequences. Here, type 1, type 4, and type 6 are the fluorescent probes provided by Affymetrix Inc., fitting the orange, green, and far red filter sets, respectively. The slides were then mounted with ProLong Gold Anti-fade Reagent with DAPI (Invitrogen) and sealed with nail polish.

### Fluorescent Immunocytochemical Staining with RNA ISH

Approximately 2 × 10^4^ NME cells from the blood of a patient or healthy donor were used for each RNA ISH slide. Approximately 2 × 10^4^ source leukocytes spiked with 5 × 10^3^ cells from a cancer cell line were applied on each control slide. After following the same protocol for RNA ISH analysis as described above without the protease-incubation and DAPI-mounting step in the manufacturer’s protocol, cells were then blocked with normal serum blocking solution (NSBS) (Table S2 in Supplementary Material) for 30 min and washed with PBS for 5 min. Subsequently, one or two primary antibodies from different species of hosts [e.g., human epidermal growth factor receptor 2 (HER2) antibody (Clone 29D8, Cell Signaling Tech), epidermal growth factor receptor (EGFR) antibody (polyclonal, Abcam), CK antibody (Clone CK3-6H5, Miltenyi Biotec), etc.] were diluted to 5 μg each antibody/ml with Normal Antibody Diluent (MP Biomedicals) and applied to the slides. The slides were then incubated for 30 min in dark at room temperature and washed three times with 1× PBS containing 0.5% Tween-20 (PBST). Each washing was approximately 5 min. The secondary antibody [e.g., Alexa Fluor (AF) 488 Donkey Anti-Rabbit IgG (H + L) (Molecular Probes), Alexa Fluor 647 Donkey Anti-Rabbit IgG (H + L) (Molecular Probes), etc.] was diluted to 5 μg each antibody/ml with PBST and applied onto the slides. The slides were incubated for 30 min in the dark at room temperature and washed three times for 5 min with PBST. The slides were then mounted with ProLong Gold Anti-fade Reagent with DAPI (Invitrogen) and sealed with nail polish.

### Microscopy Setting and Imaging

Nikon Eclipse 80i Advance Research Microscopy system was employed in this research. The Nikon Eclipse 80i Advance Research Microscope was equipped with Nikon IntensiLight C-HGFi and DS-Qi1Mc digital microscope camera. Four filter sets were used to detect and filter the signals, as shown in Table S3 in Supplementary Material. The microscopy automatic stage-moving system was designed and programed in our lab to linearly scan and capture pictures of all cells on slides. The motors were from Zaber Tech (T-LSM025A) and were controlled and automatized by the LabVIEW (National Instruments) program developed in our lab.

### Data Acquisition

The number of nucleated cells was counted before and after NME. The cell suspension was diluted at 1:25 for the cells before enrichment and at 1:10 for the cells after enrichment with 3% acetic acid. The cells in acetic acid were let stand for 10 min before counted using Fuchs-Rosenthal hemocytometer.

The enriched and stained cells on the slides were automatically counted by NIS-elements Br (Nikon Instruments Inc.) on DAPI channel. Gates for the cell circularity, size, and brightness on the nucleic acid staining were set by the NME cells from the buffy coat processed and stained in the same manner with the same protocol. The tumor-related cells that were positive on each tumor marker or combination of tumor markers were counted manually from the pictures taken by the microscopy with automatic stage-moving system.

## Results

### Immunomagnetic Negative Depletion of Patient Samples

For the blood specimens from patients with solid tumors, the average nucleated cell log_10_ depletion, the average total cell log_10_ depletion, and the average lysis efficiency were 2.65, 5.64, and 80.0%, respectively. That is, an average of more than 99.78% of nucleated blood cells was removed by our CD45-targeted NME. For the blood specimens from healthy donors, the average nucleated cell log_10_ depletion, the average total cell log_10_ depletion, and the average lysis efficiency were 2.52, 5.76, and 70.8%, respectively.

### RNA ISH on HER Markers

**Figure 2:**
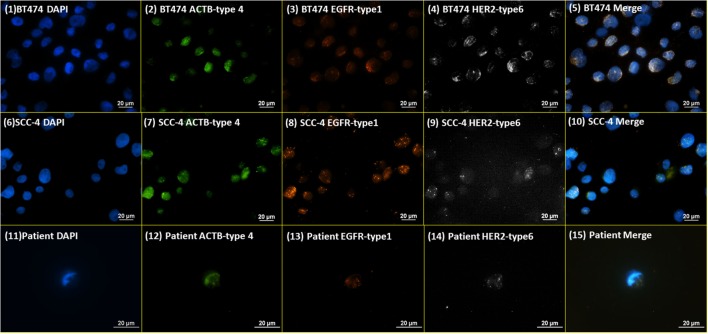
**RNA ISH test on HER family markers**. RNA ISH staining with DAPI, ACTB-type 4, EGFR-type 1, and HER2-type 6. (1)–(5) are BT474 cell line; (6)–(10) are SCC-4 cell line; (11)–(15) are the NME cells enriched from a blood specimen of a metastatic TNBC breast cancer patient.

Initially, RNA ISH was tested with the probes targeting β-actin (ACTB), HER2, and EGFR on BT474 and SCC-4 cell lines [Figure 2 (1)–(10)]. The probes targeting ACTB, EGFR, and HER2 were designed to be able to conjugate with type 4 (Green), type 1 (Orange), and type 6 (Far red) fluorescent probes, respectively. ACTB, a structural protein, of which mRNA is expressed in most human cells, is used as a housekeeping control. In the RNA ISH test on HER2 and EGFR [Figure 2 (1)–(10)], both BT474 and SCC-4 cell lines are positive for both markers, but more orange (type 1) dots are observed on SCC-4 cells than BT474 cells, while more white (type 6) dots are observed on BT474 cells than SCC-4 cells. These results indicate that SCC-4 cells show higher expression level for EGFR than BT474 cells, while BT474 cells show higher expression level for HER2 than SCC-4 cells. Qualitatively, this observation is consistent with the common results from the fluorescent ICC staining on BT474 and SCC-4. Figure 2 (11)–(15) present NME cells from a metastatic, triple-negative breast cancer (TNBC) patient, stained with the same protocol and probes. The appearance of HER2-positive cells, despite the original TNBC (negative for HER2) diagnosis of the primary tumor tissue, demonstrates the heterogeneity of CTCs. This argument is supported by the discordance in receptor status between primary tumors and CTCs or recurrent tumors in breast cancer patients as noted by others (26, 27).

### RNA ISH Specificity Test

**Figure 3:**
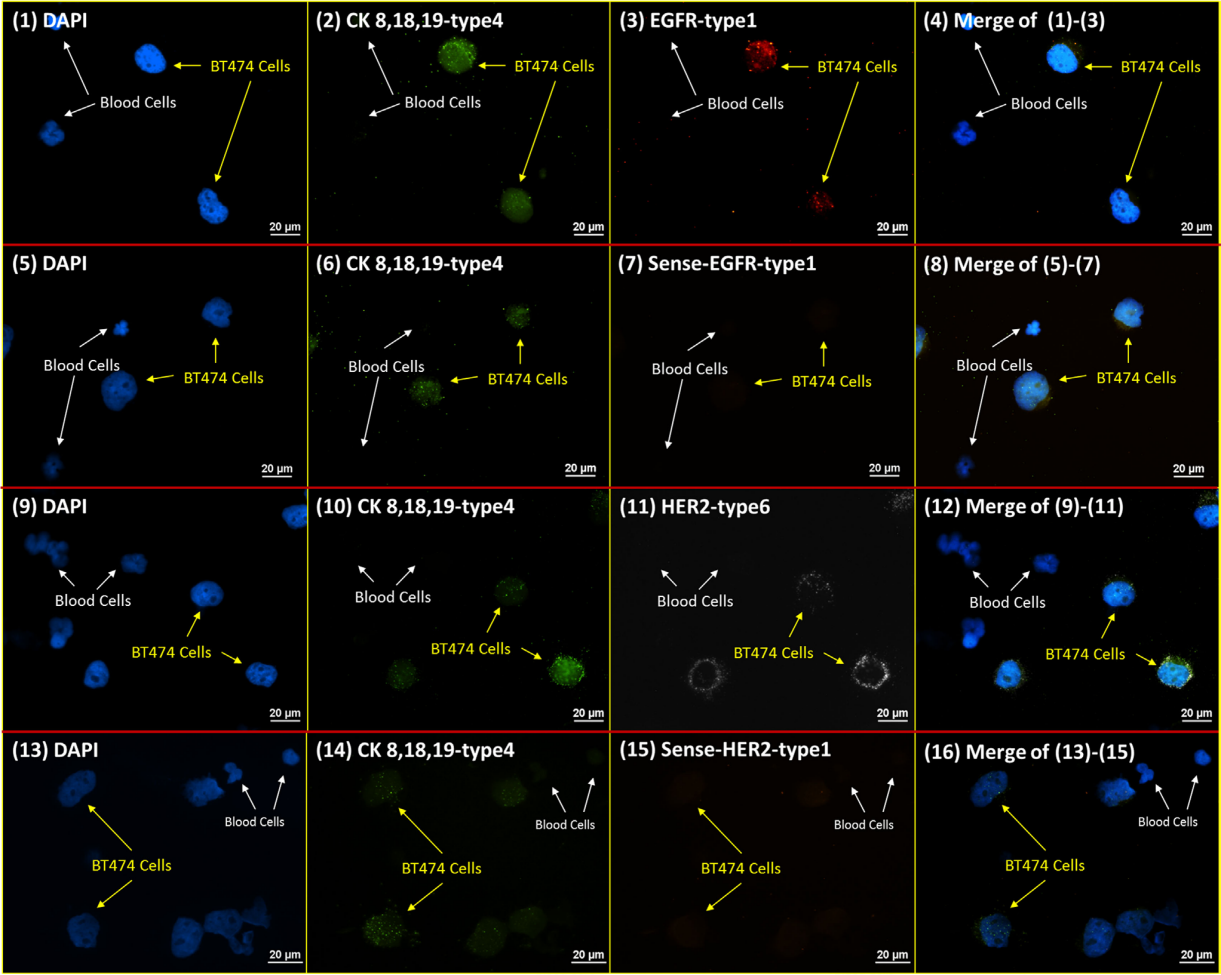
**RNA ISH specificity test**. RNA ISH specificity test with DAPI, CK 8, 18, 19-type 4, HER2 sense/anti-sense probes, and EGFR sense/anti-sense probes. The parallel stained cells are the NME cells from healthy donor spiked with BT474 cells at a ratio of 4:1 in cell number. (1)–(4) are the slide with DAPI, CK 8, 18, 19-type 4, EGFR-type 1; (5)–(8) are the slide with DAPI, CK 8, 18, 19-type 4, sense-EGFR-type 1. (9)–(12) are the slide with DAPI, CK 8, 18, 19-type 4, HER2-type 6. (13)–(16) are the slide with DAPI, CK 8, 18, 19-type 4, sense-HER2-type 1.

A measure of the specificity of RNA ISH probes was performed with customized sense probes. The sense probes were designed with the same amplification and fluorescent reporting structures as these used for the specific targeting probes (namely, anti-sense probes), but with complementary and opposite binding sequences. Theoretically, this sense probe should have zero affinity for the targeted mRNA. Figure 3 presents the RNA ISH staining with sense and anti-sense probes of HER2 and EGFR on the NME cells from healthy donor spiked with BT474 cells. Since BT474 cell line is positive on CK 8, 18, and 19, the cocktail of CK 8-type 4, CK 18-type 4, and CK 19-type 4 anti-sense probes are added simultaneously as a positive control to ensure the proper probe binding and fluorescent amplification in the RNA ISH. The results show that blood cells do not have any positive signal, while BT474 cells have positive signals only with anti-sense probes for both HER2 and EGFR. This result verifies the high specificity of RNA ISH staining.

### RNA ISH with ICC Staining Procedure Establishment Based on HER and Hedgehog Pathway Markers

**Figure 4:**
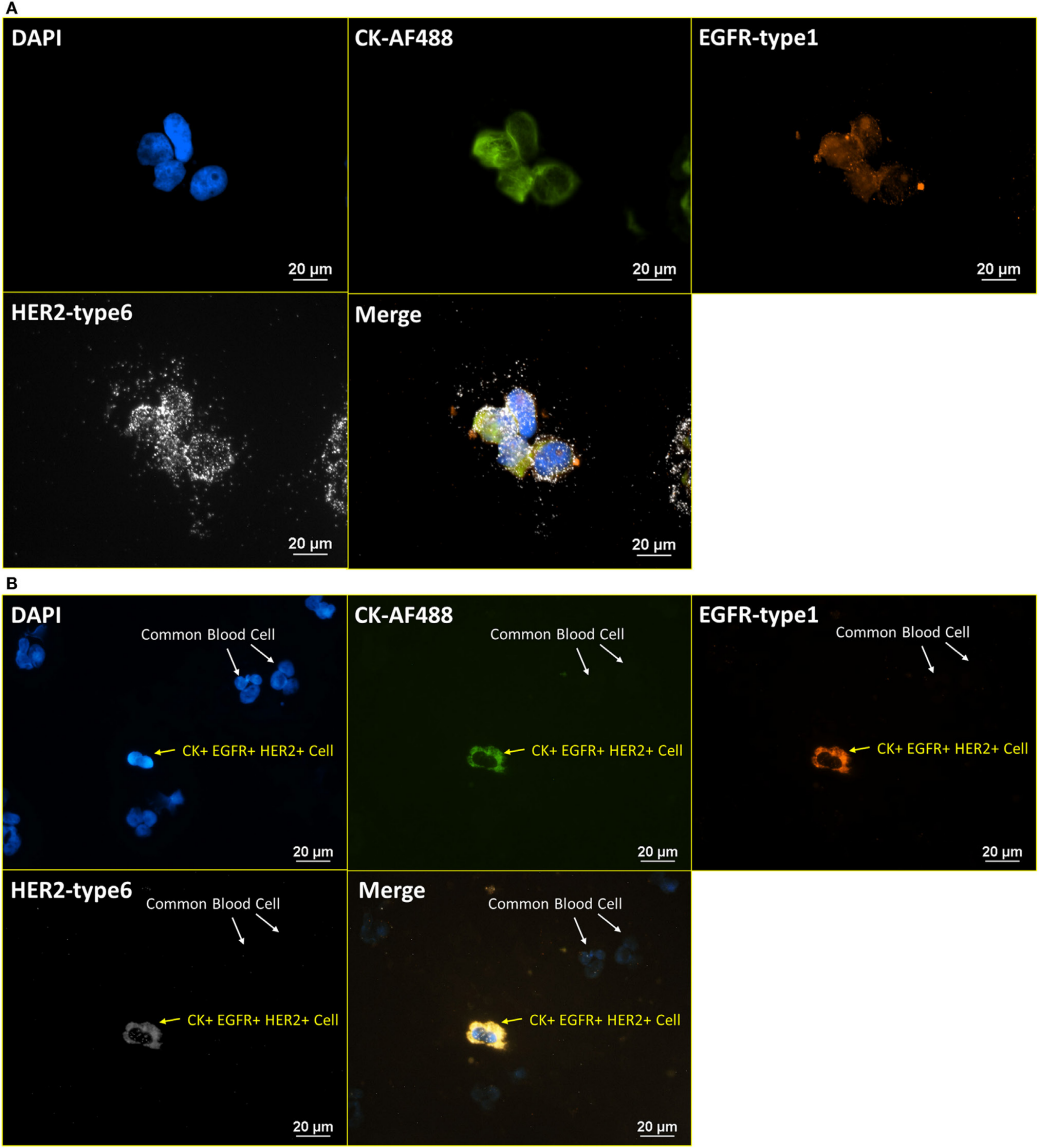
**Integrated RNA ISH and ICC staining on HER family markers**. Integrated RNA ISH and fluorescent ICC staining with customized conjugated CK-AF488 antibody and EGFR-type 1, HER2-type 6, RNA ISH. **(A)** is BT474 cells, and **(B)** is the NME cells from blood specimen of colon cancer patient. CK+, EGFR+, and HER2+ cells are found among the NME cells in the staining.

**Figure 5:**
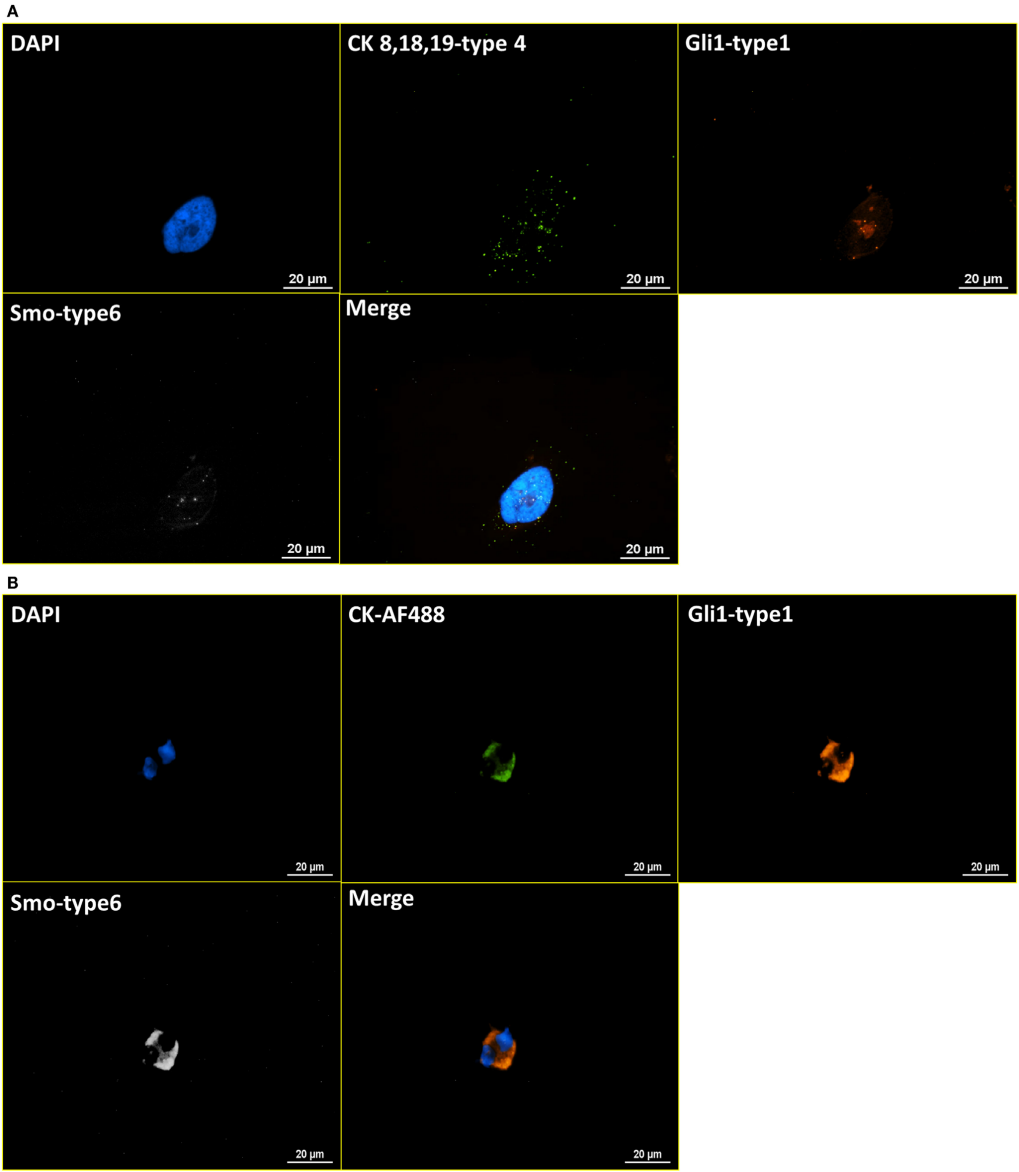
**Integrated RNA ISH and ICC staining on Hedgehog pathway markers**. RNA ISH staining with Smo-type 1 and Gli1-type 6. **(A)** is the RNA ISH staining of T47D cell line with CK 8, 18, 19-type 4, Smo-type 1, and Gli1-type 6. **(B)** is the integrated RNA ISH and fluorescent ICC staining with CK-AF488 (ICC), Smo-type 1 (RNA ISH), and Gli1-type 6 (RNA ISH) on the NME cells from a breast cancer patient. CK+, Smo+, and Gli1+ cells are found among these NME cells in the staining.

After the establishment of the protocol of RNA ISH test and the verification of specificity, the integrated RNA ISH and ICC staining procedure was developed to analyze different markers in the same cell at mRNA and protein levels. ICC staining was attempted on intracellular markers, which is usually more challenging as it requires additional permeabilization of the cell membrane that creates higher potential for non-specific bindings, comparing with ICC staining on surface markers. RNA ISH was assigned to multiple tumor markers in HER family and Hedgehog pathway to verify the robustness of RNA ISH. HER signaling is one of the most established and clinically studied pathways. And often, the status of ErbB receptor tyrosine kinase family, such as EGFR and HER2, dictates treatments for breast cancer patients. While the targeted therapies are very effective, patients may develop resistance, which results in poor outcome. Hedgehog signaling is a very valuable drug target in such case because the hedgehog molecules, such as Gli1 and Smo, are upregulated in endocrine-resistant tumors (28). Figure 4A presents the staining of DAPI, anti-CK 8, 18, 19-AF488 (ICC), EGFR-type 1 (RNA ISH), and HER2-type 6 (RNA ISH) on BT474 cells. Figure 4B is the same combination of staining applied to NME cells from a metastatic colon cancer patient. Figure 5A presents the staining of DAPI, CK 8, 18, 19-type 4, Smo-type 1, and Gli1-type 6 on T47D cells. Figure 5B is the staining of DAPI, anti-CK 8, 18, 19-AF488 (ICC), Smo-type 1, and Gli1-type 6 on the NME cells from a metastatic breast cancer patient. This results demonstrate not only the successful establishment of integrated RNA ISH and ICC staining on cell lines and patient-derived CTCs but also an evidence that the procedure can track markers from two major tumor signaling pathways (HER and Hedgehog) that are involved in many cancers.

### RNA ISH with ICC Staining – Consistency Test

**Figure 6:**
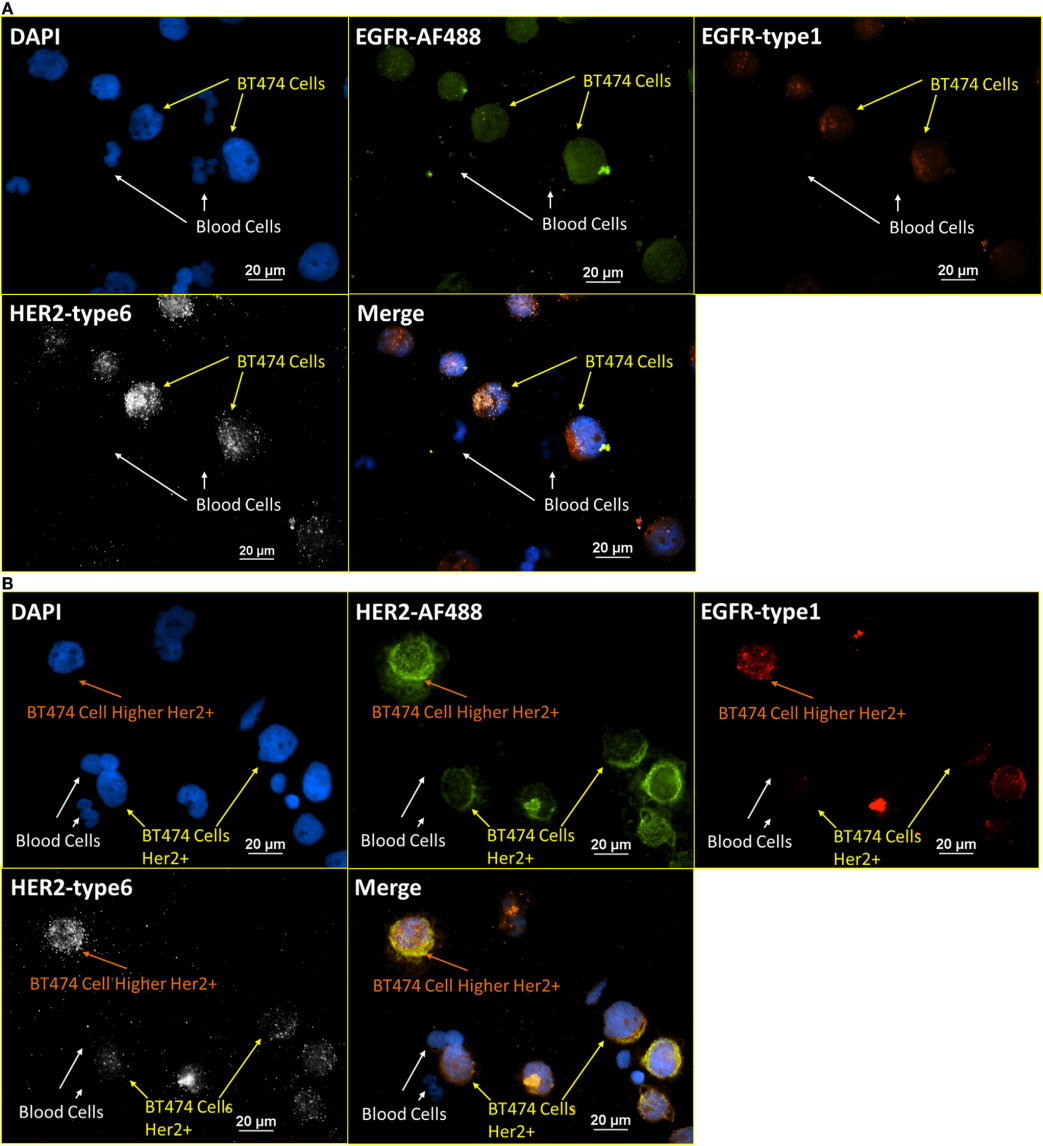
**Integrated RNA ISH and ICC staining consistency test**. RNA ISH with fluorescent ICC staining with EGFR, HER2 antibody, and EGFR, HER2 RNA ISH on blood cells spiked with BT474 cells at a ratio of 4:1 in cell number. **(A)** is the staining of DAPI, EGFR-AF488 (ICC), EGFR-type 1 (RNA ISH), and HER2-type 6 (RNA ISH). **(B)** is the staining of DAPI, HER2-AF488 (ICC), EGFR-type 1 (RNA ISH), and HER2-type 6 (RNA ISH).

To further verify the robustness of the integrated RNA ISH and ICC staining, the correlation of positive signals from RNA ISH and fluorescent ICC staining was tested. Specifically, both cell surface proteins and the corresponding mRNAs were targeted and stained on the same cell with integrated RNA ISH and ICC staining. Figure 6A presents the staining of DAPI, EGFR antibody (ICC), EGFR-type 1 (RNA ISH), and HER2-type 6 (RNA ISH) on a mixture of normal blood cells and BT474 cells (ratio of 4:1). Blood cells show no signal, while all BT474 cells are weakly positive on both EGFR fluorescent ICC staining and EGFR RNA ISH. Figure 6B presents the staining of DAPI, HER2 antibody (ICC), EGFR-type 1 (RNA ISH), and HER2-type 6 (RNA ISH). Overall, BT474 cells in Figure 6B have higher intensity of HER2 signals than those from EGFR in both RNA ISH and ICC staining. The low positivity of EGFR and high positivity of HER2 in BT474 cells from the integrated RNA ISH and ICC staining are in accordance with the previous research (29). Furthermore, in Figure 6B, BT474 cells exhibit different levels of HER2 positivity in both RNA ISH and ICC staining.

### RNA ISH with ICC Staining – Viral Targets

**Figure 7:**
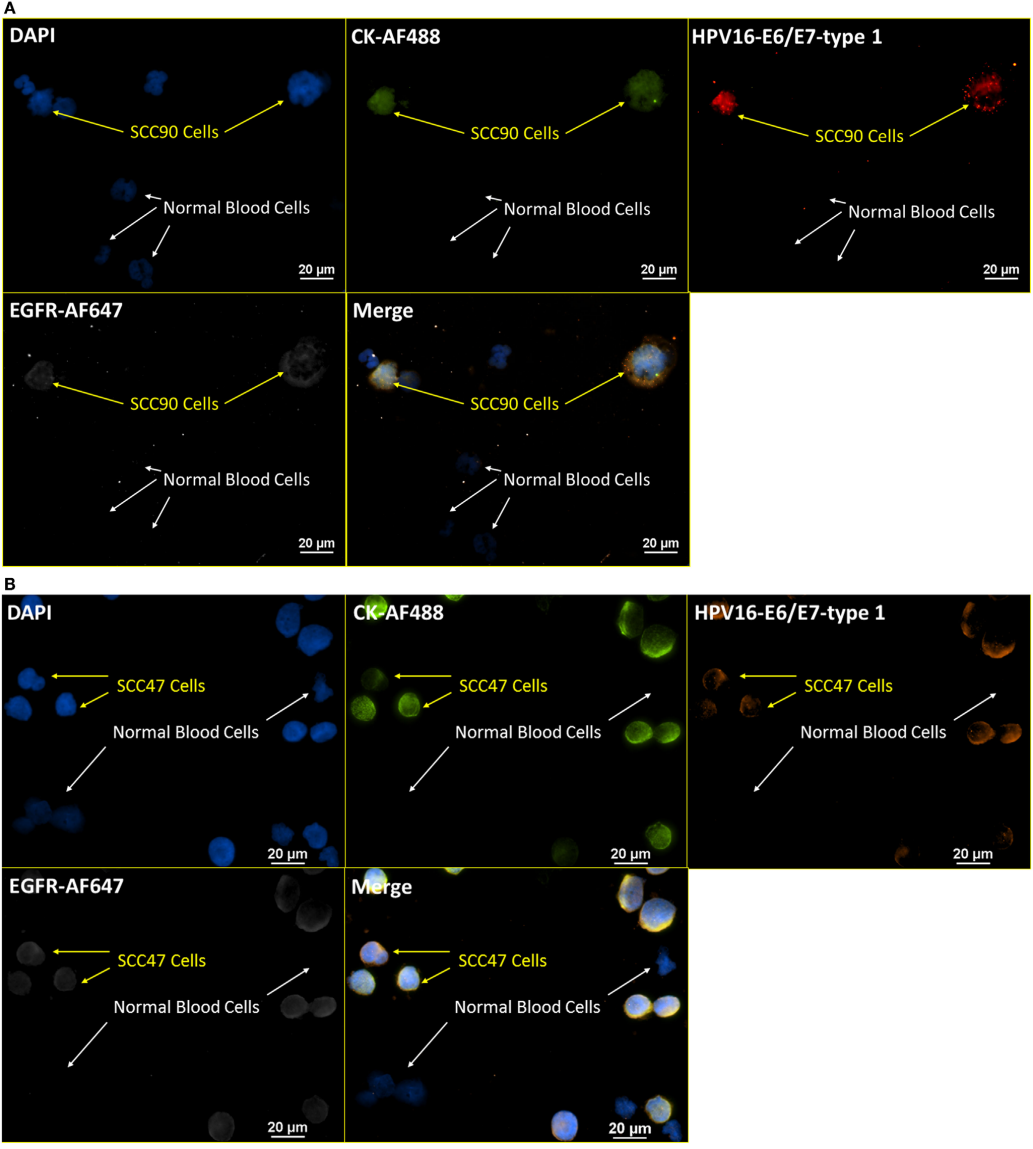

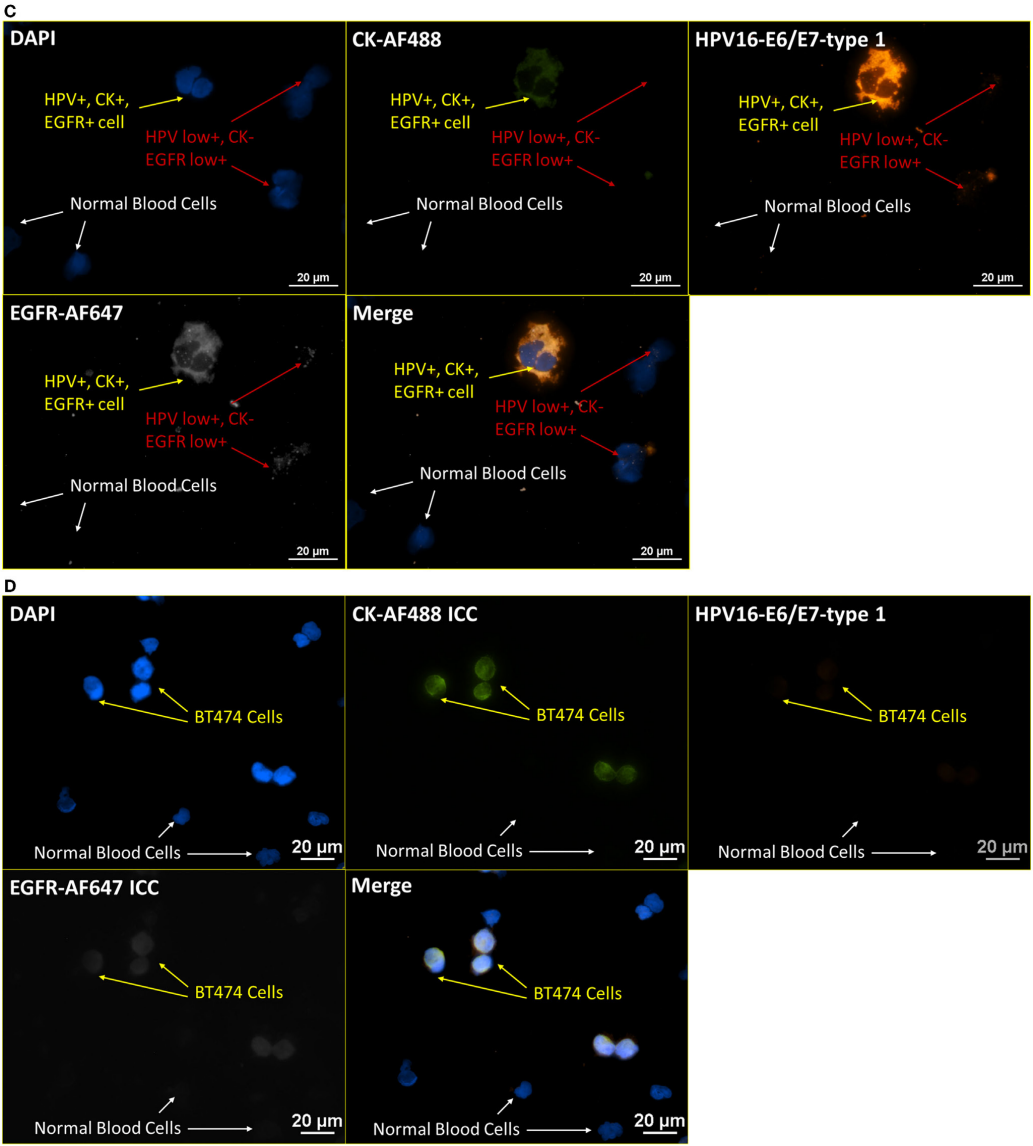
**Integrated RNA ISH and ICC staining on HPV markers**. Integrated RNA ISH and ICC staining with customized conjugated CK8, 18, 19-AF488 antibody (ICC), HPV16 E6, E7-type 1 (RNA ISH), and EGFR-AF647 antibody (ICC). The HPV16 E6, E7-type 1 probe is the 1:1 mixture of HPV16 E6-type 1 and HPV16 E7-type 1. **(A,B)** presents the staining of blood cells spiked with SCC90 **(A)** or SCC47 **(B)** cells at a ratio of 4:1 in cell number. **(C)** shows the staining of the NME cells from head and neck cancer patients. Among these NME cells shown in **(C)**, HPV+, EGFR+, CK+, or CK− cells are found. **(D)** is a specificity test with the staining of blood cells spiked with BT474 cells at a ratio of 4:1 in cell number. Both BT474 cells and blood cells are negative on HPV. The specificity of this test is hence proved.

Having verified the robustness of the incorporated RNA ISH and ICC staining on some prevailing tumor markers, we extended the application to tumor markers of different categories, including viral targets (this section) and enzymatic targets (next section). HPV16 infection is associated with the basal cells of the stratified epithelium and found in some specific types of cervical and head and neck cancers (30–32). Among the eight genes related to HPV16, E6/E7 oncogenes are associated with anti-apoptotic properties of HPV infection, while the corresponding proteins, E6/E7 proteins, are commonly used to signify the presence of HPV. To test the practicability of applying our incorporated RNA ISH and ICC staining on viral targets, a staining pattern of DAPI, CK-AF488 (ICC), HPV16 E6/E7-type 1 (RNA ISH), and EGFR-AF647 (ICC) was used on HPV-positive and -negative cell lines. Figures 7A,B are representative images from the staining of SCC90 and SCC47 cells mixed with normal blood cells (ratio of 1:4), respectively. In the RNA ISH channel of HPV16 E6/E7, the SCC47 cells have fewer dots (less than 10 dots per cell) than SCC90 (10–100 dots per cell). The observation is consistent with the results from RT-PCR and other studies, indicating that SCC90 is more positive than SCC47 in HPV16 (33, 34). Meanwhile, this staining pattern was also extended to patient samples. Figure 7C shows the parallel stained NME cells from head and neck cancer patients diagnosed with HPV-positive solid tumors. As an extended negative control, Figure 7D presents the staining with the same pattern and protocol as that applied in Figures 7A–C, on blood cells spiked with BT474 cells, an HPV-negative breast cancer cell line. The negative results in HPV16 E6/E7 channel in BT474 cells are a further check on the specificity of the HPV16 E6/E7 RNA ISH probes.

### RNA ISH with ICC Staining – Enzymatic Targets

**Figure 8:**
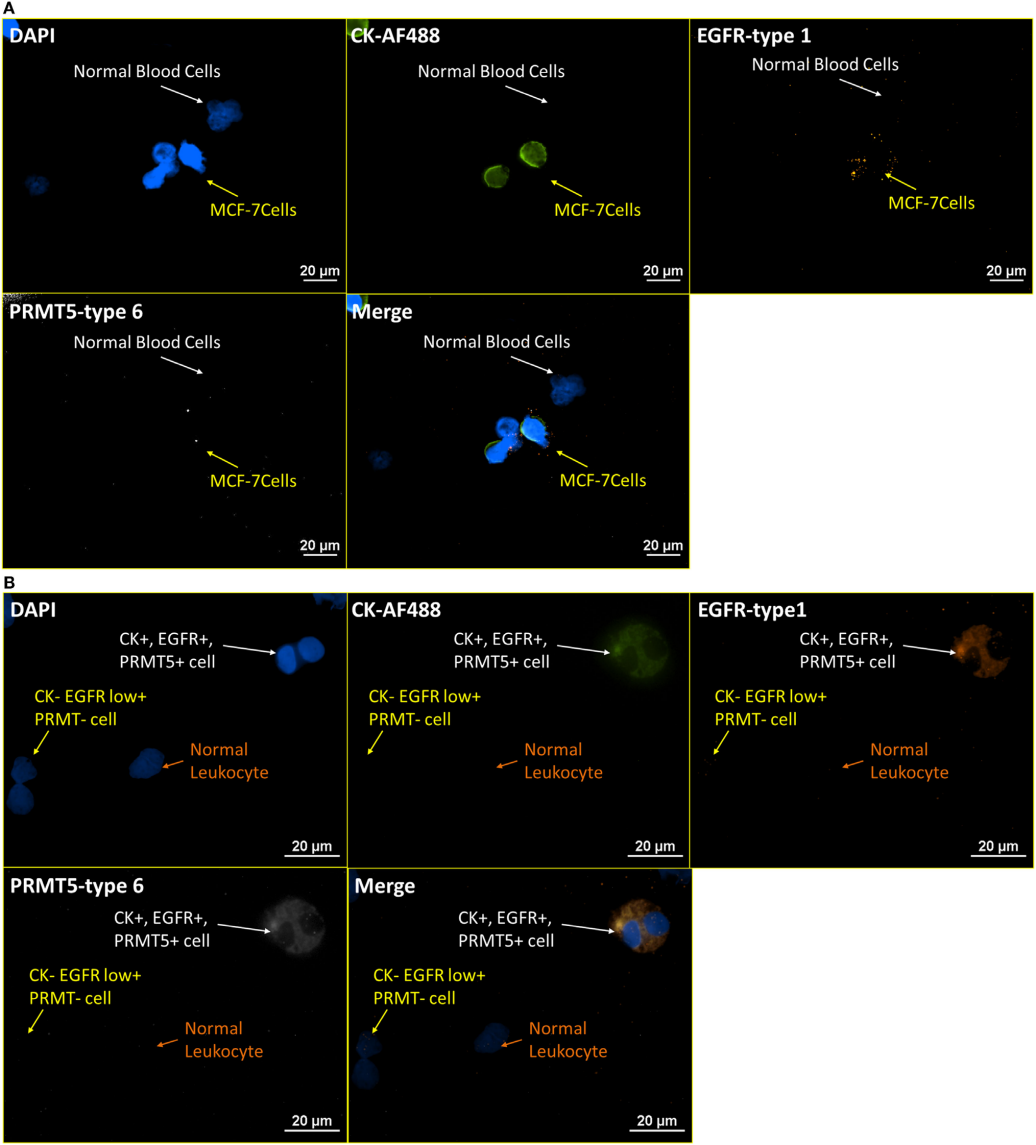
**RNA ISH test on enzymatic targets**. Integrated RNA ISH and ICC staining on enzymatic targets. Integrated RNA ISH and fluorescent ICC staining with customized conjugated CK8, 18, 19-AF488 antibody (ICC), EGFR-type 1 (RNA ISH), and PRMT5-type 6 (RNA ISH). **(A)** represents the staining of leukocytes spiked with MCF-7 cells at a ratio of 4:1 in cell number. The MCF-7 cells show low positive on PRMT5 and positive on CK and EGFR, while leukocytes are negative on all of them. **(B)** shows the staining of the NME cells from an esophageal cancer patient blood. PRMT5+ cells are found in this staining.

Labeling non-structural or membrane bound proteins, such as enzymes, in CTCs, is challenging for ICC staining, for a number of reasons, including the mobility of the proteins after cell membrane permeabilization, low protein expression level, and low specificity of antibodies. Protein arginine methyltransferase 5 (PRMT5), a Type II arginine methyltransferase, which has been reported to be involved in the regulation of multiple tumorigenesis pathways, usually can only be stained by immunohistostaining on tumor biopsy (35, 36). With the high amplification of RNA ISH and high binding specificity between complementary oligonucleotides, RNA ISH may have the potential to visualize tumor-related enzymatic proteins in CTCs. Figure 8A presents images of blood cells spiked with MCF-7 at a ratio of 4:1 that were stained for CK-AF488 (ICC), EGFR-type 1 (RNA ISH), and PRMT5-type 6 (RNA ISH). In Figure 8A, normal blood cells are negative for CK, EGFR, and PRMT5, while MCF-7 cells are positive for EGFR and weakly positive on PRMT5. Meanwhile, the same pattern was extended to the NME cells from the blood sample of an esophageal cancer patient, as presented in Figure 8B. In Figure 8B, one CTC is positive for all markers, while another cell is only weakly positive for EGFR.

## Discussion

The integrated RNA ISH and fluorescent ICC staining enables a multimodality confirmation of the positive expression of specifically targeted markers at different levels, as opposed to relying on a single antibody clone with a reported reactivity to specific isoforms of the targeted proteins. As we demonstrated in the previous study, in the prevailing ICC staining, even targeting the very common hematopoietic surface marker, CD45, can produce significantly different results depending on the selected clone of antibody and even the manufacturer where the clone is procured (10). Considering the variation in binding specificity and reactivity in regular ICC staining, the integrated RNA ISH and ICC staining may provide a more comprehensive and robust tool for visualized analysis at single-cell level for CTC research.

Exploiting the advantages of RNA ISH, several recently published studies on CTCs demonstrated the ability of RNA ISH to visualize and quantify epithelial markers, Wnt pathway markers, and CD45 (37, 38). In these studies, RNA ISH and fluorescent ICC staining were conducted on parallel slides. Consequently, while the presence of individual markers can be determined *via* these approaches, the heterogeneity of CTCs prevents the conclusive determination of various marker combinations on a cell-by-cell basis. To eliminate the errors from CTC heterogeneity in parallel RNA ISH and fluorescent ICC staining, the integrated RNA ISH and fluorescent ICC staining provides a solution to get information from both RNA ISH and ICC staining on the same cells.

To further investigate the specificity and selectivity of our RNA ISH technology, various combinations of cell types, probes, antibodies, and targeting markers were tested in this research. Unlike antibodies that frequently have low level of non-specific binding, especially on blood samples, we did not observe any positive signals in the cell types that were assumed to be negative for the specially targeted RNA ISH probes. The sense controls presented in Figure 3 further confirmed that the RNA ISH probes were highly selective and specific. The high specificity of RNA ISH was also supported by Figures 4–8 in which no RNA ISH-positive signal was detected from the blood cells that were present, either through spiking or in the case of patient samples, from the blood cells that made through the magnetic depletion step. As a further demonstration of the specificity of the RNA ISH staining, Figures 6A,B demonstrated that the cells stained with the antibodies targeting EGFR or HER2 were also positive with the RNA ISH targeting the appropriate mRNA; meanwhile, the blood cells spiked with the cell lines were negative for both the antibodies and RNA ISH.

The RNA ISH technology in this study creates a signal amplification tree using branched single-stranded DNA. This amplification tree can provide 400 binding sites for each labeling probe binding to single target sequence on mRNA. Several fluorescent molecules can be bound to each binding site and thus achieve a theoretical 8000-fold amplification for each mRNA target sequence bound. It has been reported that the high selectivity and high amplification levels in this RNA ISH probe design enable the tracking of single transcript of target mRNA or microRNA, as well as the quantitative analysis of the transcription level of the targeting gene. This quantitative analysis is precise enough to create a dynamic profile on the synthesis, transportation, and degradation of the nascent mRNAs after a chemical induction on the transcription of the corresponding targeted gene (19). Some later reports developed a computational identification procedure with multiple, singly labeled probes to count the RNA transcripts automatically (39). Here, in this study, we have not yet determined how many mRNAs correspond to each bright spot, which is an ongoing focus in our laboratory.

Despite the merits and superiority of RNA ISH over ICC staining in specificity, reactivity, and quantitation as discussed above, ICC staining, which targets proteins, may provide more direct information of CTCs since protein is the functional unit in most signal pathways. In the common practice of tumor cell line analysis, RNA ISH and ICC staining are applied to support each other on parallel slides from the same batch of cells of certain tumor cell lines. However, we suggest that unlike the relatively homogeneous tumor cell lines, the highly heterogeneous CTCs require all staining markers in all staining methods on the same cells. Our integrated RNA ISH and ICC staining can fulfill this challenging requirement and provide an analytical method for comprehensive visualized labeling on different markers in tumor-related pathways.

A challenge with this RNA ISH technology combined with ICC is exposure time. Since for a specific marker, the amount of mRNA is usually lower than the amount of the corresponding protein, the signal from RNA ISH is usually weaker (although presumably more specific and precise) than that from ICC staining. Correspondingly, the camera exposure time for RNA ISH staining under the fluorescent microscopy scanning is about 5–20 times longer than that for ICC staining. Hence, in a visualized fluorescent characterization of the enriched CTCs which inevitably have some healthy, irrelevant cells, the automated fluorescent microscopy scanning takes 5–20 times longer to detect and analyze the target cells on the slides with RNA ISH staining than those with ICC staining. One solution in using an integrated RNA ISH and ICC staining protocol might consist of an automated fluorescent microscopy scanning system to quickly search the tumor-related target cell populations by ICC staining. Once identified, these targeted cells could then be analyzed for RNA ISH staining. This is part of the reason why the selection of the ICC markers in our integrated RNA ISH and ICC staining are usually prevailing tumor markers with high expression, such as CK and HER family markers.

For the ease of the access and better resolution in our current presentation, instead of confocal imaging which typically uses laser light, we chose to use a traditional epi-fluorescence microscope with excitation and emission filters, dichroic mirrors, and an intensified mercury bulb. This approach requires longer exposure time, accompanied with the increased potential of the signal “leaking” from the other ICC dyes into the RNA ISH channel. For example, it is suggested that weak and diffuse orange color in the EGFR-type 1 images in Figures 6A,B is leakage from the AF488 signals, but not the actual RNA ISH signals which are typically observed as bright spots (i.e., Figure 2). To address this issue, future studies will use a Nuance spectral deconvolution system, which allows the “leaking signals” to be subtracted from the corrected images.

In some images from RNA ISH slides (i.e., Figure 6), despite the careful optimization on the fixing time with 4% paraformaldehyde and protease treatment, bright spots indicating positive signals can be observed outside of the cells. Initially, these outlying spots were regarded as non-specific binding to background impurities. However, in the staining of both sense/anti-sense controls (Figure 3) and BT474 cells which serve as negative control for E6/E7 staining of HPV16 infected cells (Figure 7D), there were no bright spots near tumor cells. One hypothesis for these positive spots outside of the cells is the leakage of mRNA fragments. The RNA ISH staining protocol requires intensive permeabilization to facilitate the delivery of nucleic acid probes, amplifiers, and fluorochromes across the cell membrane. Thus, it is possible that some cell contents leak out of cells during the permeablization step. Another hypothesis is that these bright, extracellular spots are extracellular vesicles. The extracellular vesicles secreted from some cancer cell lines have been found to contain many tumor-related mRNA sequences *via* RT-PCR and RNA microarrays (40, 41). Further work is needed to examine the hypotheses and better understand the phenomenon.

## Conclusion

The research aims to provide a robust separation and characterization method for heterogeneous CTCs. The CTCs were initially enriched by our NME process, in which all blood cells were concentrated and labeled with CD45 antibody that is conjugated with magnetic beads. The labeled cells then flowed through our customized QMS system where the non-hematopoietic cells without CD45-magnetic beads labeling were enriched. To compliment prevailing ICC staining, we introduced RNA ISH into our CTC characterization method and evaluated its specificity and sensitivity. Furthermore, the integrated RNA ISH and ICC staining was developed to allow visualized multiparameter analysis at both RNA and protein levels.

The integrated RNA ISH and fluorescent ICC staining provides an approach to study the heterogeneity of CTCs by incorporating the strengths of antibody labeling and RNA ISH. To verify the practicability and robustness of this integrated staining, tumor markers of different categories, including epithelial markers, HER family markers, Hedgehog pathway markers, viral markers, enzymatic markers, etc., were tested on both cell line and NME cells from healthy donors and patients.

Currently, in our lab, the high expressing or well-studied tumor markers are stained with ICC staining, which has higher signal intensity, to shorten the duration of automated detection of CTCs. Meanwhile, RNA ISH is applied on novel markers to perform visualized, specific, and precise detection. The ongoing work in our laboratory is focused on the use of spectral deconvolution technology not only to sharpen the images for each fluorescent signal but also to extend the number of targets stained with both ICC and RNA ISH technology up to seven distinct markers as well as the detailed quantitative evaluation of the staining results.

## Author Contributions

YW, K-JJP, CD, PA, and BM conducted the experiments. QP, MZ, ML, and JC designed the experiments. YW, K-JJP, and JC wrote the text.

## Conflict of Interest Statement

The authors declare that the research was conducted in the absence of any commercial or financial relationships that could be construed as a potential conflict of interest.

## References

[1] van de Stolpe A Pantel K Sleijfer S Terstappen LW den Toonder JM. Circulating tumor cell isolation and diagnostics: toward routine clinical use. Cancer Res (2011) 71(18):5955–60. 10.1158/0008-5472.CAN-11-1254 21896640

[2] Alix-Panabières C Pantel K. Technologies for detection of circulating tumor cells: facts and vision. Lab Chip (2014) 14:57–62. 10.1039/c3lc50644d 24145967

[3] Riethdorf S Fritsche H Müller V Rau T Schindlbeck C Rack B Detection of circulating tumor cells in peripheral blood of patients with metastatic breast cancer: a validation study of the CellSearch system. Clin Cancer Res (2007) 13(3):920–8. 10.1158/1078-0432.CCR-06-1695 17289886

[4] Powell AA Talasaz AH Zhang H Coram MA Reddy A Deng G Single cell profiling of circulating tumor cells: transcriptional heterogeneity and diversity from breast cancer cell lines. PLoS One (2012) 7(5):e33788. 10.1371/journal.pone.0033788 22586443 PMC3346739

[5] Yu M Bardia A Wittner BS Stott SL Smas ME Ting DT Circulating breast tumor cells exhibit dynamic changes in epithelial and mesenchymal composition. Science (2013) 339(6119):580–4. 10.1126/science.1228522 23372014 PMC3760262

[6] Zhang L Ridgway LD Wetzel MD Ngo J Yin W Kumar D The identification and characterization of breast cancer CTCs with brain metastatic competence. Sci Transl Med (2013) 5(180):84–93. 10.1126/scitranslmed.3005109 PMC3863909 23576814

[7] Lustberg MB Balasubramanian P Miller B Garcia-Villa A Deighan C Wu Y Heterogeneous atypical cell populations are present in blood of metastatic triple negative breast cancer. Breast Cancer Res (2014) 16:R23 10.1186/bcr3622 24602188 PMC4053256

[8] Lara O Tong X Zborowski M Chalmers JJ Enrichment of rare cancer cells through depletion of normal cells using density and flow-through, immunomagnetic cell separation. Exp Hematol (2004) 32(10):891–904. 10.1016/j.exphem.2004.07.007 15504544

[9] Balasubramanian P Lang JC Jatana KR Miller B Ozer E Old M Multiparameter analysis, including EMT markers, on negatively enriched blood samples from patients with squamous cell carcinoma of the head and neck. PLoS One (2012) 7(7):e42048. 10.1371/journal.pone.0042048 22844540 PMC3406036

[10] Wu Y Deighan CJ Miller BL Balasubramanian P Lustberg MB Zborowski M Isolation and analysis of rare cells in the blood of cancer patients using a negative depletion methodology. Methods (2013) 64(2):169–82. 10.1016/j.ymeth.2013.09.006 24056212 PMC3874448

[11] Theodoropoulos PA Polioudaki H Agelaki S Kallergi G Saridaki Z Mavroudis D Circulating tumor cells with a putative stem cell phenotype in peripheral blood of patients with breast cancer. Cancer Lett (2010) 288(1):99–106. 10.1016/j.canlet.2009.06.027 19619935

[12] Kallergi G Agelaki S Kalykaki A Stournaras C Mavroudis D Georgoulias V. Phosphorylated EGFR and PI3K/Akt signaling kinases are expressed in circulating tumor cells of breast cancerpatients. Breast Cancer Res (2008) 10(5):R80. 10.1186/bcr2149 18822183 PMC2614515

[13] Buchwalow I Samoilova V Boecker W Tiemann M Non-specific binding of antibodies in immune-histochemistry: fallacies and facts. Sci Rep (2011) 1:28 10.1038/srep00028 22355547 PMC3216515

[14] Smirnov DA Zweitzig DR Foulk BW Miller MC Doyle GV Pienta KJ Global gene expression profiling of circulating tumor cells. Cancer Res (2005) 65(12):4993–7. 10.1158/0008-5472.CAN-04-4330 15958538

[15] O’Hara SM Moreno JG Zweitzig DR Gross S Gomella LG Terstappen LW. Multigene reverse transcription-PCR profiling of circulating tumor cells in hormone-refractory prostate cancer. Clin Chem (2004) 50(5):826–35. 10.1373/clinchem.2003.028563 14988224

[16] Sieuwerts AM Mostert B Bolt-de Vries J Peeters D de Jongh FE Stouthard JM mRNA and microRNA expression profiles in circulating tumor cells and primary tumors of metastatic breast cancer patients. Clin Cancer Res (2011) 17(11):3600–18. 10.1158/1078-0432.CCR-11-0255 21505063

[17] Zhao L Lu YT Li F Wu K Hou S Yu J High-purity prostate circulating tumor cell isolation by a polymer nanofiber-embedded microchip for whole exome sequencing. Adv Mater (2013) 25:2897–902. 10.1002/adma.201205237 23529932 PMC3875622

[18] Kang JH Krause S Tobin H Mammoto A Kanapathipillai M Ingber DE. A combined micromagnetic-microfluidic device for rapid capture and culture of rare circulating tumor cells. Lab Chip (2012) 12(12):2175–81. 10.1039/c2lc40072c 22453808

[19] Chen XX Bai F. Single-cell analyses of circulating tumor cells. Cancer Biol Med (2015) 12(3):184–92. 10.7497/j.issn.2095-3941.2015.0056 26487963 PMC4607822

[20] Femino AM Fay FS Fogarty K Singer RH. Visualization of single RNA transcripts in situ. Science (1998) 280(5363):585–90. 10.1126/science.280.5363.585 9554849

[21] Taniguchi Y Choi PJ Li GW Chen H Babu M Hearn J Quantifying *E. coli* proteome and transcriptome with single-molecule sensitivity in single cells. Science (2010) 329(5991):533–8. 10.1126/science.1188308 20671182 PMC2922915

[22] Itzkovitz S van Oudenaarden A. Validating transcripts with probes and imaging technology. Nat Methods (2011) 8(4 Suppl):S12–9. 10.1038/nmeth.1573 21451512 PMC3158979

[23] White JS Weissfeld JL Ragin CC Rossie KM Martin CL Shuster M The influence of clinical and demographic risk factors on the establishment of head and neck squamous cell carcinoma cell lines. Oral Oncol (2007) 43(7):701–12. 10.1016/j.oraloncology.2006.09.001 17112776 PMC2025692

[24] Yang L Lang JC Balasubramanian P Jatana KR Schuller D Agrawal A Optimization of an enrichment process for circulating tumor cells from the blood of head and neck cancer patients through depletion of normal cells. Biotechnol Bioeng (2009) 102(2):521–34. 10.1002/bit.22066 18726961 PMC3906726

[25] Garcia-Villa A Balasubramanian P Miller BL Lustberg MB Ramaswamy B Chalmers JJ Assessment of γ-H2AX levels in circulating tumor cells from patients receiving chemotherapy. Front Oncol (2012) 2:128 10.3389/fonc.2012.00128 23112954 PMC3480704

[26] Fehm T Hoffmann O Aktas B Becker S Solomayer EF Wallwiener D Detection and characterization of circulating tumor cells in blood of primary breast cancer patients by RT-PCR and comparison to status of bone marrow disseminated cells. Breast Cancer Res (2009) 11(4):R59. 10.1186/bcr2349 19664291 PMC2750121

[27] Aurilio G Disalvatore D Pruneri G Bagnardi V Viale G Curigliano G A meta-analysis of oestrogen receptor, progesterone receptor and human epidermal growth factor receptor 2 discordance between primary breast cancer and metastases. Eur J Cancer (2014) 50:277–89. 10.1016/j.ejca.2013.10.004 24269135

[28] Ramaswamy B Lu Y Teng KY Nuovo G Li X Shapiro CL Hedgehog signaling is a novel therapeutic target in tamoxifen-resistant breast cancer aberrantly activated by PI3K/AKT pathway. Cancer Res (2012) 72(19):5048–59. 10.1158/0008-5472.CAN-12-1248 22875023 PMC3837449

[29] Subik K Lee JF Baxter L Strzepek T Costello D Crowley P The expression patterns of ER, PR, HER2, CK5/6, EGFR, Ki-67 and AR by immunohistochemical analysis in breast cancer cell lines. Breast Cancer (Auckl) (2010) 4:35–41. 10.4137/BCBCR.S0 20697531 PMC2914277

[30] Horvath CA Boulet GA Renoux VM Delvenne PO Bogers JP. Mechanisms of cell entry by human papillomaviruses: an overview. Virol J (2010) 7:11. 10.1186/1743-422X-7-11 20089191 PMC2823669

[31] Pett M Coleman N. Integration of high-risk human papillomavirus: a key event in cervical carcinogenesis? J Pathol (2007) 212:356–67. 10.1002/path.2192 17573670

[32] Pytynia KB Dahlstrom KR Sturgis EM. Epidemiology of HPV-associated oropharyngeal cancer. Oral Oncol (2014) 50(5):380–6. 10.1016/j.oraloncology.2013.12.019 24461628 PMC4444216

[33] Kimple RJ Smith MA Blitzer GC Torres AD Martin JA Yang RZ Enhanced radiation sensitivity in HPV-positive head and neck cancer. Cancer Res (2013) 73(15):4791–800. 10.1158/0008-5472.CAN-13-0587 23749640 PMC3732540

[34] Olthof NC Huebbers CU Kolligs J Henfling M Ramaekers FC Cornet I Viral load, gene expression and mapping of viral integration sites in HPV16-associated HNSCC cell lines. Int J Cancer (2015) 136:e207–18. 10.1002/ijc.29112 25082736 PMC5370555

[35] Bao X Zhao S Liu T Liu Y Liu Y Yang X. Overexpression of PRMT5 promotes tumor cell growth and is associated with poor disease prognosis in epithelial ovarian cancer. J Histochem Cytochem (2013) 61(3):206–17. 10.1369/0022155413475452 23292799 PMC3636695

[36] Nicholas C Yang J Peters SB Bill MA Baiocchi RA Yan F PRMT5 is upregulated in malignant and metastatic melanoma and regulates expression of MITF and p27^Kip1^. PLoS One (2013) 8(9):e74710 10.1371/journal.pone.0074710 24098663 PMC3786975

[37] Yu M Ting DT Stott SL Wittner BS Ozsolak F Paul S RNA sequencing of pancreatic circulating tumour cells implicates WNT signalling in metastasis. Nature (2012) 487(7408):510–3. 10.1038/nature11217 22763454 PMC3408856

[38] Payne R Wang F Su N Krell J Zebrowski A Yagüe E Viable circulating tumour cell detection using multiplex RNA in situ hybridization predicts progression-free survival in metastatic breast cancer patients. Br J Cancer (2012) 106(11):1790–7. 10.1038/bjc.2012.137 22538972 PMC3364118

[39] Raj A van den Bogaard P Rifkin SA van Oudenaarden A Tyagi S. Imaging individual mRNA molecules using multiple singly labeled probes. Nat Methods (2008) 5(10):877–9. 10.1038/nmeth.1253 18806792 PMC3126653

[40] Jenjaroenpun P Kremenska Y Nair VM Kremenskoy M Joseph B Kurochkin IV. Characterization of RNA in exosomes secreted by human breast cancer cell lines using next-generation sequencing. PeerJ (2013) 1:e201. 10.7717/peerj.201 24255815 PMC3828613

[41] Chiba M Kimura M Asari S. Exosomes secreted from human colorectal cancer cell lines contain mRNAs, microRNAs and natural antisense RNAs, that can transfer into the human hepatoma HepG2 and lung cancer A549 cell lines. Oncol Rep (2012) 28(5):1551–8. 10.3892/or.2012.1967 22895844 PMC3583404

